# A Terahertz CMOS *V*-Shaped Patch Antenna with Defected Ground Structure

**DOI:** 10.3390/s18082432

**Published:** 2018-07-26

**Authors:** Hyeongjin Kim, Wonseok Choe, Jinho Jeong

**Affiliations:** Department of Electronic Engineering, Sogang University, 35 Baekbeom-ro, Mapo-gu, Seoul 04107, Korea; ilyrs486@gmail.com (H.K.); rhuphy@naver.com (W.C.)

**Keywords:** antenna, CMOS, defected ground structure, patch, terahertz

## Abstract

In this paper, a *V*-shaped patch antenna with defected ground structure is proposed at terahertz to overcome the limited performance of a standard complementary metal-oxide semiconductor (CMOS) patch antenna consisting of several metal layers and very thin interdielectric layers. The proposed *V*-shaped patch with slots allows the increased radiation resistance and broadband performance. In addition, the patch resonating at different frequency from the *V*-shaped patch is stacked on the top to broaden the impedance-matching bandwidth. More importantly, the slots are formed in the ground plane, which is called the defected ground structure, to further increase the radiation resistance and thus improve the bandwidth and efficiency. It is verified from electromagnetic simulations that the leakage waves from the defected ground can enhance the antenna directivity and gain by coherently interfering with the topside radiation. The proposed on-chip antenna is fabricated using a standard 65 nm CMOS process. The on-wafer measurement shows very wide bandwidth in input reflection coefficient (<−10 dB), greater than 28.7% from 240 to >320 GHz. The measured peak gain was as high as 5.48 dBi at 295 GHz. To the best of the authors’ knowledge, these results belong to the best performance among the terahertz CMOS on-chip antennas without using additional components or processes such as dielectric resonators, lens, or substrate thinning.

## 1. Introduction

Recently, there has been active research on terahertz (THz) communication, sensing, and imaging systems using semi-conductor transistor technologies such as silicon (Si) complementary metal-oxide semiconductor (CMOS) field effect transistors (FETs), gallium arsenide (GaAs) or indium phosphide (InP) heterojunction bipolar transistors (HBTs), and high-electron mobility transistors (HEMTs) [1,2,3,4]. The THz monolithic integrated circuits (TMICs) that use these technologies allow for lower cost, higher integration, and miniaturization of THz systems, compared with optics-based components [5,6,7,8,9,10].

THz antennas with high radiation efficiency and broad bandwidth are indispensable in the front-end stage of TMICs for communication, sensing, and imaging systems. Basically, off-chip antennas can provide broadband and high efficiency performance by using low-loss substrates such as low-temperature co-fired ceramic (LTCC) and artificial dielectrics [11,12]. However, parasitic components such as bonding wires and interconnection lines used in the connection of TMICs and off-chip antenna can result in the severe performance degradation (impedance mismatch and insertion loss), poor repeatability, and increased cost.

On the contrary, on-chip antennas can effectively avoid these drawbacks by eliminating off-chip bonding wires and interconnection lines [13]. Especially, the antenna size can be drastically reduced due to the very short wavelengths at THz frequencies, so that the antenna can be integrated onto Si CMOS ICs. Therefore, the on-chip antenna approach allows low-cost and repeatable THz solutions for mass production. Generally, CMOS on-chip antennas with front-side radiation are designed using several metal and interlayer dielectric layers, where the bottom metal layer is used as a ground plane to shield the high-conductivity Si substrate and minimize dielectric loss [14,15]. In this case, the metallic ground plane in proximity to the top patch creates an image problem and cancels out the radiated field by the patch. A metamaterial or reactive impedance surface (RIS) technique can be used to replace the metallic ground plane in order to mitigate this problem and enhance the bandwidth and efficiency [16,17,18]. Anyway, a very thin dielectric between the radiating element and ground in CMOS on-chip antenna results in low radiation resistance and thus narrow bandwidth and poor efficiency, both around 10% [13,19] at the THz frequency.

Several approaches have been introduced to improve the bandwidth and efficiency of the CMOS on-chip antenna. In [20], a dielectric resonator was stacked on the on-chip patch antenna providing an excellent efficiency of 80% with a bandwidth of 11% at 344 GHz in a 130 nm CMOS process. However, this 3-dimensional antenna requires an additional process to adhere the etched dielectric onto the fabricated antenna using an epoxy glue in a high precision.

Back-side radiating antennas through the Si substrate were also proposed, showing high gain and wideband performance [1,21]. In this case, the electromagnetic (EM) wave can be absorbed by a high-conductivity and high-dielectric constant Si substrate in a substrate mode. In order to reduce the loss due to the substrate mode, the substrate is thinned to less than 100 μm [22]. In addition, a hemispherical high-resistivity Si lens is attached to the backside of the substrate to increase the gain [23]. Several back-side radiating CMOS on-chip antennas have been proposed at a THz frequency. In [24,25], a broadband bowtie antenna array was used for a THz imager in low-cost CMOS technology, where there was no ground plane, and the bowtie antenna was made up of all the metal layers. The high-resistivity Si substrate was ground down to 130 μm in order to reduce the substrate loss. In [26], artificial magnetic conductors (AMCs) were adopted in the BiCMOS bowtie antenna to stop the wave from radiating through the lossy Si substrate, where AMCs function as a high impedance surface. It was shown from the simulation that the bowtie antenna can achieve about 1 dB higher gain (~−1.9 dB) by adopting an AMC structure at 94 GHz. However, substrate thinning and the use of a lens can decrease the reliability and increase the packaging cost.

In this paper, we propose a *V*-shaped patch antenna using a defected ground structure (DGS) operating at *H*-band (220–320 GHz), to enhance the efficiency and bandwidth of a standard CMOS patch antenna. In addition, the influence of the probe tip used in on-wafer antenna measurement is analyzed through an EM simulation. Section 2 discusses the efficiency and bandwidth limitations of a standard CMOS on-chip patch antenna. The *V*-shaped patch antenna with DGS is proposed and designed in the same section, including the EM analysis of the probe effect on the antenna performance. The measurement results are presented in Section 3. Finally, Section 4 presents the conclusion.

## 2. CMOS On-Chip Patch Antenna Design

### 2.1. Standard CMOS On-Chip Patch Antenna

Microstrip patch antennas are widely used as THz Si CMOS on-chip antennas because of their simple structure, front-side radiation, and easy integration with other transceiver circuits. In the CMOS patch antenna, the high conductivity (σ=10 S/m) and high dielectric constant ($\varepsilon_{r}=11.9$) of the Si substrate is generally shielded by using the bottom metal layer as a ground plane. Therefore, one of upper metal layers is selected as a radiating patch and thus the interlayer dielectric (SiO_2_) serves as a substrate of the patch antenna. In the conventional sub-μm CMOS process, the available thickness of the SiO_2_ ($\varepsilon_{r}=4.3$) layer is less than a few tenths of a micrometer. This reduces the radiation resistance of the patch antenna, which leads to severe degradation in radiation efficiency and bandwidth.

**Figure 1:**
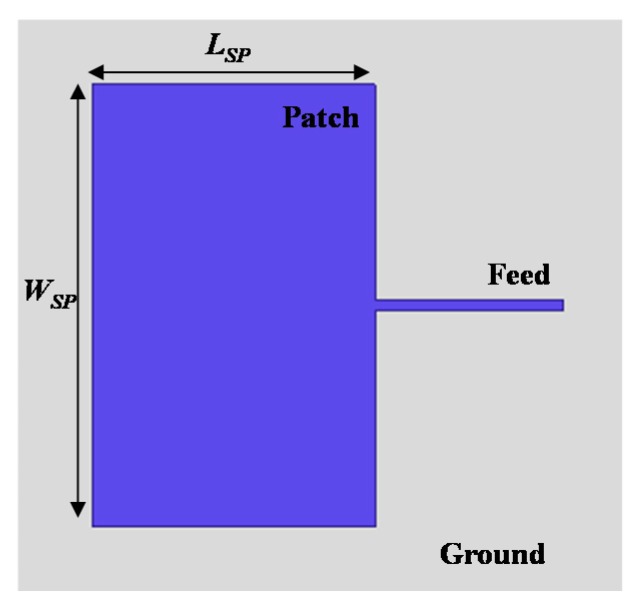
Top view of THz on-chip patch antenna.

**Figure 2:**
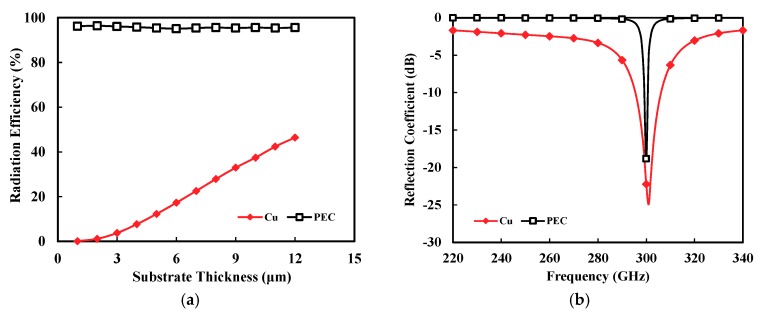
Simulation of the standard CMOS patch antenna. (**a**) Radiation efficiency according to substrate thickness (at a fixed frequency of 300 GHz). (**b**) Reflection coefficient (|*S*_11_|) with frequency (*W_SP_* = 345 μm, *L_SP_* = 225 μm, and substrate thickness of 4.49 μm).

In order to investigate the effect of the substrate thickness, a standard CMOS patch antenna was designed at 300 GHz, as shown in Figure 1. It consisted of very thin SiO_2_ dielectric layer as a substrate. Figure 2 shows the simulation results of a designed patch antenna by using a commercial full-wave simulator (Ansoft High Frequency Structure Simulator (HFSS)). If metal layers were set to a perfect electric conductor (PEC), the standard patch maintained a very high radiation efficiency of around 96.1% when the substrate thickness varied from 1 to 12 μm, as shown in Figure 2a. In this simulation, the loss tangent of the substrate was assumed to be 0.001 for simulation purposes [27]. However, the efficiency rapidly dropped for copper (Cu) metal layers, because of its finite conductivity ($\sigma =5.2\times 10^{7}\;S/m$). Furthermore, the efficiency dramatically degraded from 37.5 to 12.3% as the substrate thickness decreased from 10 to 5 μm. Note that the patch size was varied in the simulation to maintain the same resonance frequency depending on the substrate thickness. The radiation efficiency in the simulation was the ratio of total radiated power ($P_{rad}$) to source-unavailable power ($P_{avs}$), but with input power ($P_{in}$), or $\eta_{in}=P_{rad}/P_{in}$. That is, the simulated radiation efficiency ($\eta_{in}$) in this paper did not take into account the antenna input mismatch (or input reflection coefficient, $\left|S_{11}\right|$). A more practical definition of the efficiency is $\eta_{avs}=\frac{P_{rad}}{P_{avs}}=\left(1-{\left|S_{11}\right|}^{2}\right)\eta_{in}$. The simulated performance of the designed antenna is presented in the paper using radiation efficiency ($\eta_{in}$) and input reflection coefficient ($\left|S_{11}\right|$).

Figure 2b shows that the standard CMOS patch exhibited a very narrow input reflection coefficient ($20\log\left|S_{11}\right|$) due to a very thin substrate thickness of 4.49 μm. That is, the fractional bandwidth of the input reflection coefficient <−10 dB was just 0.3 and 3.0% for the PEC and Cu patch, respectively.

**Figure 3:**
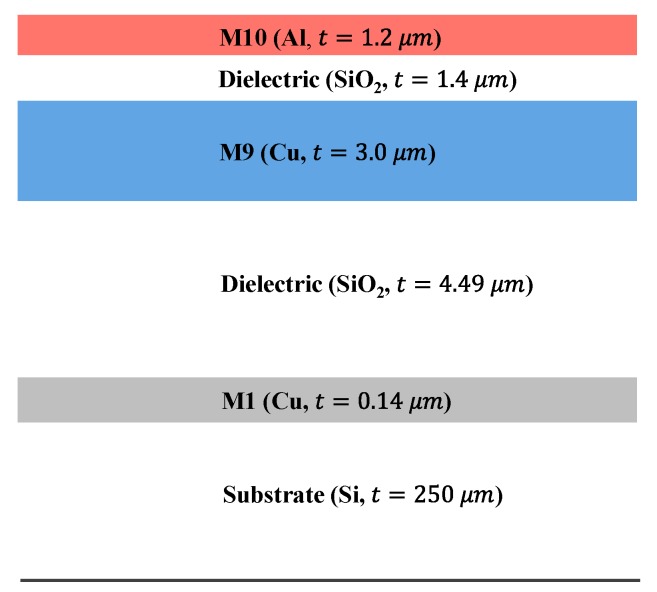
Cross-sectional view of the 65 nm Si CMOS process.

In summary, the standard CMOS patch antenna exhibited very low efficiency and narrowband performance, which were caused by low radiation resistance and high conductor loss mainly due to a very thin substrate. In this work, we proposed a V-shaped patch antenna with DGS to improve the efficiency and bandwidth of the CMOS patch antenna. Figure 3 shows the metal/dielectric layer structure in a commercial 65 nm CMOS process used for the on-chip antenna design in this work. It consisted of a total of 10 metal layers with dielectric (SiO_2_) interlayers. The top metal layer, M10 in aluminum (Al), is generally used as bias lines in IC. Note that M9 (Cu) had a higher conductivity and higher thickness than M10 (Al). In the patch antenna design, therefore, the M9 layer is selected instead of M10 as a radiating patch, in order to reduce the conductor loss. The other metal layers (M1–M8) in Cu were very thin (0.1–0.3 μm), resulting in high conductor loss.

### 2.2. V-Shaped Patch Antenna

**Figure 4:**
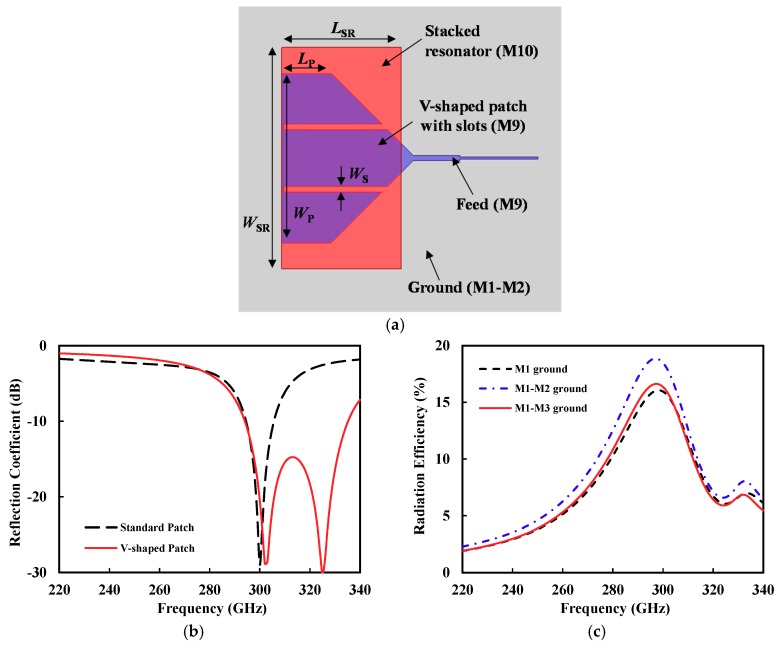
(**a**) Top view of the proposed *V*-shaped patch antenna (*W_P_* = 325 μm, *L_P_* = 95 μm, *W_S_* = 10 μm, *W_SR_* = 425 μm, *L_SR_* = 230 μm, feed width/length = 9 μm/90 μm, 50 Ω-line width = 4 μm). (**b**) Simulated reflection coefficient (|*S*_11_|). (**c**) Simulated efficiency according to the metal layer composition of the ground plane.

Figure 4a shows the proposed *V*-shaped patch with M9 and M1 layers as the patch and ground, respectively. The *V*-shaped patch can increase the radiation resistance compared with the rectangular patch, while the slots formed in the patch improving the matching bandwidth [28,29]. The *V*-shaped patch was designed to have a resonant frequency of around 300 GHz. In addition, a rectangular resonator (M10) was stacked on the patch. Its size was determined to provide a resonant frequency (around 325 GHz) that was different from the M9 *V*-shaped patch (around 300 GHz), to broaden the matching bandwidth. The proposed patch antenna exhibited a bandwidth of 15.5%, which was 12.5% higher than the standard patch antenna, as shown in Figure 4b. The bandwidth in this paper was defined as the frequency band meeting the input reflection coefficient ($20\log\left|S_{11}\right|$) of lower than −10 dB. The simulated directivity was 7.6 dBi and the gain was −0.4 dBi, at 300 GHz.

In the above simulation, M1 was only used as a ground plane to obtain the highest SiO_2_ substrate. However, M1 was very thin (0.14 μm-thick) so that the conductor loss in the ground plane could be very large. If more metal layers are used as a ground plane, the conductor loss can be reduced. Figure 4c shows the simulated radiation efficiency of the *V*-shaped patch antenna according to the metal layer composition of the ground plane. The ground plane, consisting of M1 and M2, allowed for the best efficiency performance, or a 4% increase at 300 GHz compared with the M1-only ground. On the other hand, when all M1, M2, and M3 layers were used as a ground, and the conductor loss increased due to the decrease of the dielectric thickness. Therefore, the M1-M2 ground plane was used in the patch antenna.

### 2.3. V-Shaped Patch Antenna with a Defected Ground Structure

**Figure 5:**
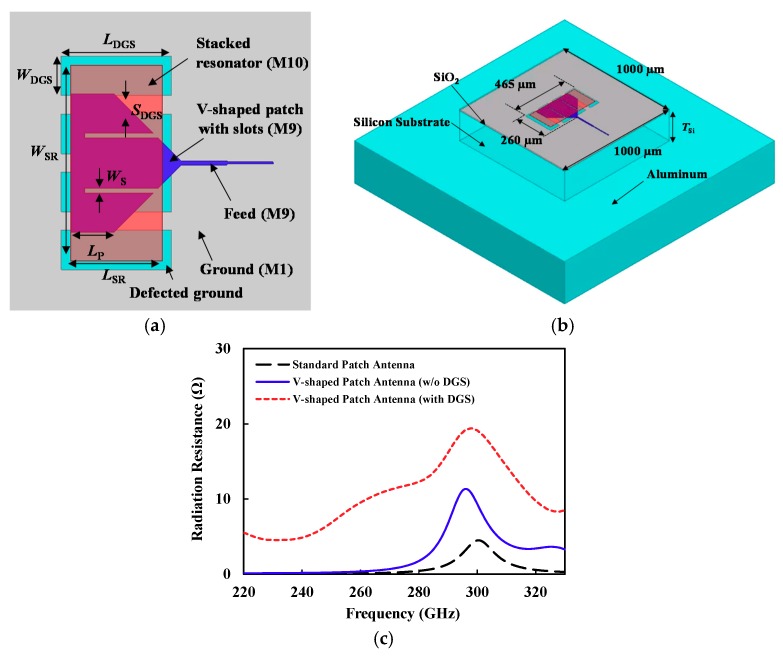
(**a**) Top view of the proposed *V*-shaped patch antenna with a defected ground structure (DGS); (**b**) HFSS simulation model of the proposed on-chip antenna, including the backside metal (aluminum); (**c**) Simulated radiation resistance of the patch antennas.

As stated above, a very thin dielectric in a CMOS on-chip patch antenna reduces the radiation resistance leading to low efficiency and narrow bandwidth. In order to increase the radiation resistance, we proposed the defected ground structure (DGS), in which the repetitive slots are formed in the ground plane, as shown in Figure 5a,b. These slots reduce the capacitance between the radiating patch and the ground, resulting in an increase in radiation resistance. Figure 5c compares the simulated radiation resistance of the standard patch and *V*-shaped patch without DGS, and the *V*-shaped patch with DGS, showing that *V*-shape and DGS effectively increased the radiation resistance across a wide bandwidth.

**Figure 6:**
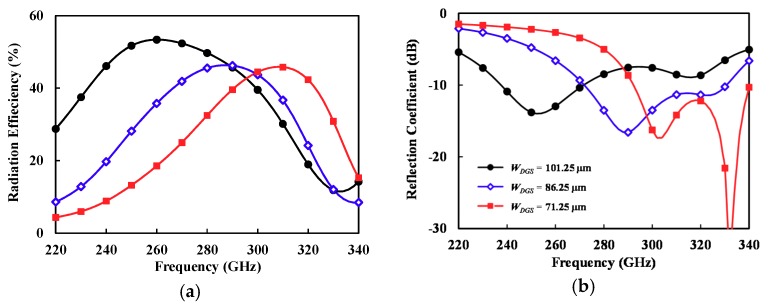
Simulation results of the V-shaped patch antenna with DGS depending on the slot width (W_DGS_). (**a**) Efficiency. (**b**) Reflection coefficient (|*S*_11_|).

However, the DGS allows some parts of the EM wave to leak into the lossy Si substrate, which can increase the dielectric loss. Therefore, the slot size (width $W_{DGS}$, length $L_{DGS}$, and spacing $S_{DGS}$) was optimized for maximum efficiency through EM simulation of the structure shown in Figure 5b. Figure 6 shows the simulated efficiency and reflection coefficient of the *V*-shaped patch antenna with DGS as a function of slot width ($W_{DGS}$), where the total width ($4\times W_{DGS}+3\times S_{DGS}$) and length $L_{DGS}$ are fixed to 465 and 240 μm, respectively. The slot width $W_{DGS}$ of 101.25 μm presents a maximum efficiency of 52% at 260 GHz as shown in Figure 6a. However, the −10 dB reflection coefficient bandwidth was not the best, and our target frequency was around 300 GHz. Thus, we selected $W_{DGS}$ of 86.25 μm, which allowed for a maximum efficiency of 46% at 295 GHz and a bandwidth of 15.2%. All the antenna dimensions were optimized considering bandwidth and efficiency at a center frequency of 300 GHz. Table 1 lists the optimized antenna parameters. Note that the wavelength in the air was 1 mm at 300 GHz.

**Figure 7:**
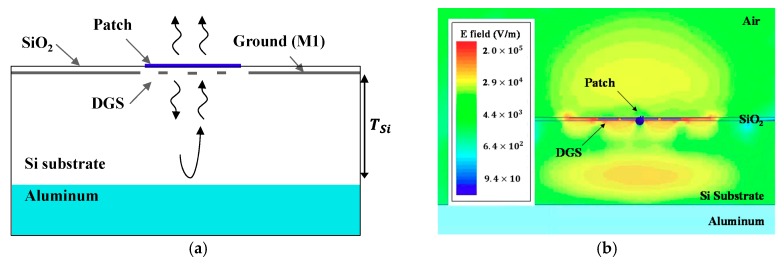
(**a**) Diagram of radiating waves in the proposed antenna. (**b**) E-field distribution of the proposed antenna.

The patch antenna basically generates front-side radiation as illustrated in Figure 7a. The back-side radiation from the patch could leak into the Si substrate through the slots on the ground plane. Some part of the leakage wave was dissipated in the lossy Si substrate in the form of a surface wave. The remaining wave was reflected by the backside ground plane (aluminum in Figure 7a) and returned to the patch, generating interference with the front-side radiating wave. Figure 7b shows the electric field distribution of the designed *V*-shaped patch antenna with DGS on the 250 μm-thick Si substrate at 300 GHz. There is a relatively strong leakage wave inside the Si substrate. Actually, if thickness of Si substrate ($T_{Si}$) satisfied (1), the two waves (front-side radiating wave and the reflected wave by the backside ground) added in phase and increased both the directivity and the gain of the antenna:

(1) $$T_{Si}=\left(2k+1\right)\frac{\lambda_{g}}{4},\;\left(k=0,1,2,3,\cdots \right),$$

where $\lambda_{g}$ is a guided wavelength in the silicon and is about 290 μm at 300 GHz.

**Figure 8:**
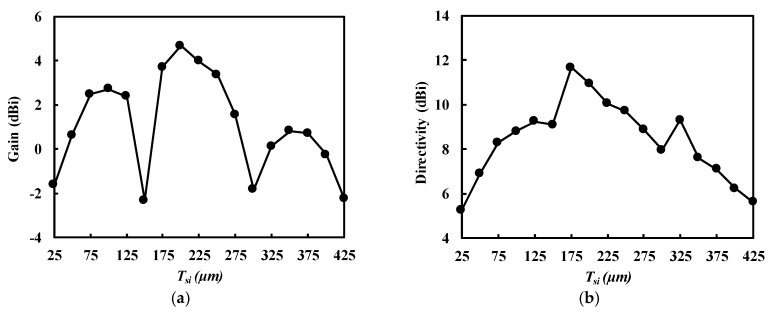
Simulated gain with respect to the thickness of Si substrate (*T_Si_*). (**a**) Gain. (**b**) Directivity.

Figure 8 shows the simulated gain and directivity of the V-shaped patch antenna with DGS at 300 GHz, depending on $T_{Si}$. The gain is maximized around $T_{Si}$ = 72.5 μm ($\lambda_{g}/4$) and 217 μm ($3\lambda_{g}/4$) and minimized around $T_{Si}\;$= 145 μm ($\lambda_{g}/2$) and 290 μm ($\lambda_{g}$), respectively, as expected from (1). With a substrate thicker than 300 μm, the leakage wave could propagate through the Si substrate guided by the M1–M2 and backside ground planes (like parallel-plate waveguide), so that the gain rapidly decreased, as shown in Figure 8 [30]. The Si substrate used in this work had $T_{Si}$ = 250 μm, which corresponded to 0.86$\lambda_{g}$ close to $3\lambda_{g}/4$. It led to the additive interference and an increase in directivity and gain. The designed *V*-shaped patch antenna with DGS resulted in a high directivity of 9.72 dB and a high gain of 3.37 dB at 300 GHz.

### 2.4. Probe Effect on the Antenna Performance

The performance of the fabricated on-chip antenna, such as input reflection coefficient, gain, and radiation pattern, was measured by on-wafer probing. The metallic probe was placed 300 μm (0.3$\lambda_{0}$ at 300 GHz) apart from the radiating antenna, so that it could affect the radiation performance [31]. In other words, the probe tip reflects the EM wave radiated by the on-chip antenna, leading to the distortion of antenna radiation pattern [32].

**Figure 9:**
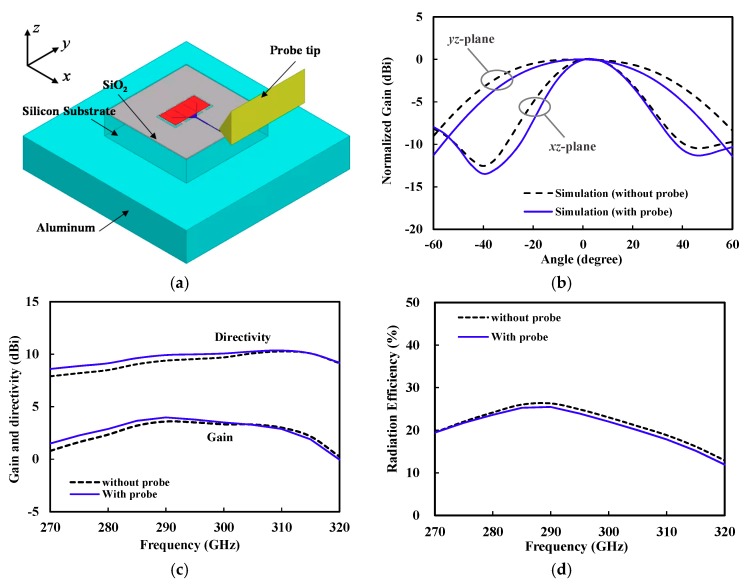
(**a**) Electromagnetic (EM) simulation of the on-chip antenna with the probe tip included. (**b**) Simulated radiation pattern at 300 GHz. (**c**) Simulated gain and directivity. (**d**) Simulated radiation efficiency.

In this work, we performed EM simulation of the on-chip antenna with the probe tip included, as shown in Figure 9a. For the simplicity of the simulation, the probe tip was modeled as a gold triangular prism. Figure 9b shows the simulated radiation patterns (with and without probe) at 300 GHz. The simulation including the probe, exhibited a narrower beam-width in both *E*- and *H*-planes. This result implicated that the probe used in the on-wafer measurement could create the wave reflections and result in higher directivity [33]. Figure 9c,d show that the directivity and gain could be increased by 0–0.7 dB at a frequency range from 270 to 300 GHz, and the radiation efficiency was rather reduced by 0–1% due to the probe. The simulation showed that the probe had a negligible effect on the input impedance match.

## 3. Measurement

### Measurement of the On-Chip Antenna

**Figure 10:**
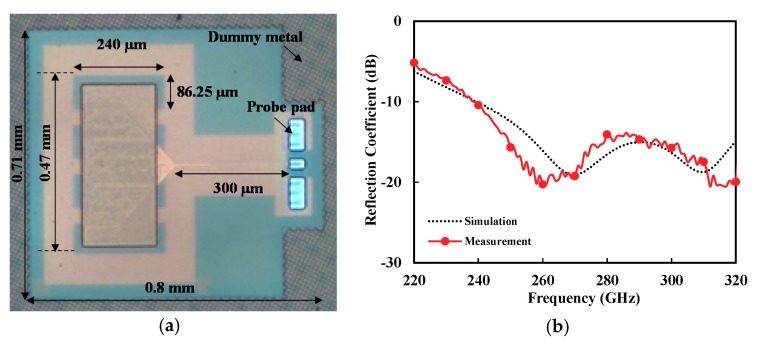
(**a**) Photograph of the fabricated on-chip antenna. (**b**) Measured input reflection coefficient of the proposed antenna.

The designed *V*-shaped patch antenna with DGS was fabricated using a commercial 65 nm CMOS process. Figure 10a shows the photograph of the fabricated antenna. The chip size was 0.71 mm × 0.74 mm, including the radio frequency (RF) probe pad.

Figure 10b shows the measured reflection coefficient of the fabricated on-chip antenna at H-band, using a vector network analyzer and WR-03 frequency extenders. It exhibited a very wide band performance, from 240 to >320 GHz (fractional bandwidth >28.7%), with a reflection coefficient better than −10 dB. It is also shown that the simulation agreed well with the measurement.

**Figure 11:**
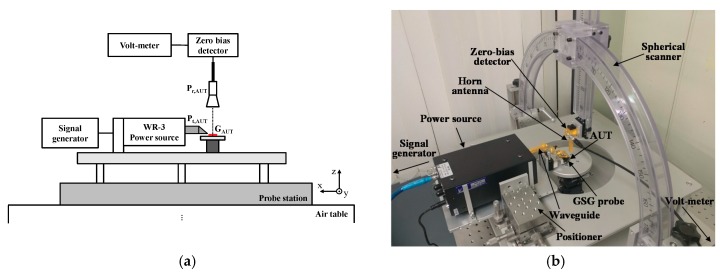
THz on-chip antenna measurement setup. (**a**) Diagram. (**b**) Photograph.

The gain and radiation pattern were measured by on-wafer probing using the THz on-chip antenna measurement setup developed by the authors, as shown in Figure 11. An *H*-band signal was generated using a signal generator (12.2–18.4 GHz) and frequency multiplier chains (×18), and it was applied to the antenna under test (AUT) through the probe. The radiated power from the AUT was measured by the detector with a standard horn antenna that was mounted on the spherical scanner. Therefore, the radiation pattern could be measured by manually changing the angle of the detector in the *E-* and *H-*planes.

The gain of AUT ($G_{AUT}$) was measured based on the gain comparison method [34,35]. That is, the received power ($P_{r,AUT}$) was measured using the setup shown in Figure 11. Then, the AUT was replaced with a standard horn antenna with a known gain ($G_{horn}$). The received power ($P_{r,horn}$) was compared with $P_{r,AUT}$ to determine ($G_{AUT}$) as follows:

(2) $$G_{AUT}=P_{r,AUT}-P_{r,horn}+G_{horn}+L_{probe},$$

where $L_{probe}$ is the loss of the probe.

**Figure 12:**
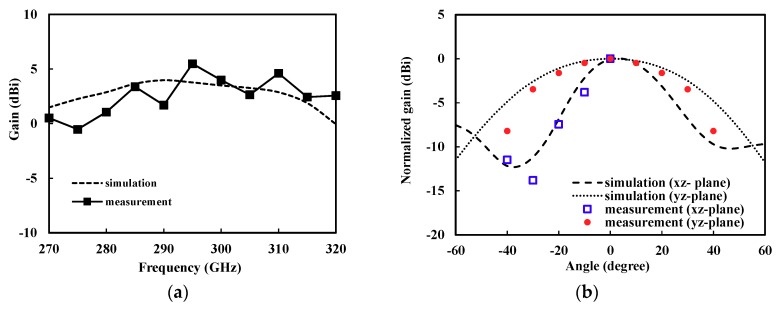
Measured performance of the fabricated antenna. (**a**) Gain as a function of frequency; (**b**) Radiation pattern at a peak gain frequency (295 GHz). Simulation results include the probe tip.

Figure 12a shows the measured gain of the antenna with frequency. It was higher than about 15 dBi from 280 to 320 GHz with a peak gain of 5.48 dBi at 295 GHz. Figure 12b shows the measured radiation pattern at a peak gain frequency of 295 GHz. Both figures show close agreement with the simulation results.

The reported THz CMOS on-chip antennas are compared in Table 2. The proposed *V*-shaped patch antenna in this work exhibited the best bandwidth performance (meeting reflection coefficient lower than −10 dB). Furthermore, the gain was the best among on-chip antennas without using additional external components or processes such as dielectric resonators [20], lens [23], or wafer thinning [22]. Note that most publications in this table do not provide the measurement results of the on-chip antenna, because of the difficulties of the THz on-chip antenna measurement. 

## 4. Conclusions

In this paper, a front-side radiating CMOS patch antenna was studied to enhance bandwidth and efficiency. Several ideas were proposed to overcome the problems caused by a very thin dielectric substrate in the standard CMOS process. At first, the standard patch antenna was modified for wide impedance matching bandwidth by proposing the slotted *V*-shaped patch with an extra resonator stacked on top. Then, a defected ground structure was proposed to increase the radiation resistance for high efficiency and wide bandwidth. The simulation verified that the leakage wave in Si substrate through the slots in the ground plane can be added in phase with the front-side radiating wave from the patch, leading to an enhancement in directivity and gain. In addition, it was also proved by the simulation that the probe used in the on-wafer measurement can affect the radiation pattern of the on-chip antenna.

The fabricated *V*-shaped CMOS on-chip antenna was measured using the developed THz antenna measurement setup. It exhibits a very wide bandwidth (greater than 28.7%) in the input reflection coefficient. The peak gain was 5.48 dBi at 295 GHz. Therefore, the developed high-gain broadband antenna can be integrated in terahertz CMOS transceiver circuits for communications and imaging applications.

## Figures and Tables

**Table 1:** Design parameters of the proposed on-chip antenna.

Parameter	*L_SR_*	*L_P_*	*W_SR_*	*W_P_*	*W_S_*	*L_DGS_*	*W_DGS_*	*S_DGS_*
Value (μm)	200	95	425	300	5	240	86	40

**Table 2:** Comparison of the reported Si CMOS on-chip antennas operating around 300 GHz.

	This Work	[1]	[36]	[37]	[38]	[39]	[20]	[23]
Technology	65 nm	65 nm	130 nm	40 nm	65 nm	32 nm	130 nm	65 nm
Center frequency (GHz)	290	240	280	340	270	210	340	288
Bandwidth (%) ^1^	>28	8.3 *	2.1 *	4.1 *	12 *	20 *	11	n.a.
Peak efficiency (%)	26.3 *	11.4 *	21 *	6.5 *	21.4 *	24 *	80	65
Peak gain (dBi)	5.48	1.55 *	−1.6 *	−5.5 *	−0.5 *	−2.5 *	10	n.a.
Type	Patch	Slotted loop	Patch	Patch	SIW ^2^	Dipole	DRA ^3^	HHLA ^4^
Planar	Yes	Yes	Yes	Yes	Yes	Yes	No	No

* Simulation result; ^1^ Bandwidth of input reflection coefficient <−10 dB; ^2^ Substrate-integrated-waveguide; ^3^ DRA: dielectric resonator antenna; ^4^ HHLA: hyper-hemispherical lens antenna.
